# Murine liver response to *Allium sativum* treatment during infection induced-trypanosomiasis

**DOI:** 10.1016/j.sjbs.2021.02.067

**Published:** 2021-03-01

**Authors:** Mohamed A. Dkhil, Esam M. Al-Shaebi, Ahmed S. Alazzouni, Saleh Al-Quraishy, Mona Khalil

**Affiliations:** a Department of Zoology, College of Science, King Saud University, Saudi Arabia; b Department of Zoology and Entomology, Faculty of Science, Helwan University, Egypt

**Keywords:** Trypanosomiasis, Mice, Liver, *Allium sativum*

## Abstract

Hepatic injury induced by trypanosomiasis is one of the major health problems not only to human but also to wild and domestic animals. This study aimed to evaluate the hepatoprotective role of *Allium sativum* extract (ASE) against *Trypanosoma evansi* infection in mice. Animals were divided into 4 groups. Group I received only saline while group II received ASE (20 mg/Kg). Animals of group III and group IV were infected with *T. evansi*. The latter group was treated with ASE. The infrared spectroscopic analysis of *A. sativum* extract exhibited bands between 3700 cm^−1^ and 599 cm^−1^. On day 4 post *T. evansi* infection, ASE decreased the parasitemia by about 15 fold. Also, ASE regulated the number of erythrocytes and leucocytes and the hemoglobin content. In addition, the histopathological damage was reduced after treatment with ASE. Moreover, the oxidant and the antioxidant markers (glutathione, malondialdehyde and catalase) were regulated in the infected-treated animals. Collectively, the results proved the protective role of ASE against *T. evansi* infection in mice.

## 1 Introduction

Trypanosomiasis, a vector-borne disease induced by a parasitic protozoon of the genus *Trypanosoma*. It affects several populations worldwide especially in Africa (Mehlhorn 2014). Not only human but also wild and domestic animals could be infected with Trypanosomes leading to anemia, fever, weakness and weight loss (Otto et al., 2009).The transmission of the disease from a host to another occurs via the horsefly, a blood sucking insect.

Trypanosomiasis caused by trypanosomes can affect humans and animals, and about 57 million people are at risk of infection (Franco et al., 2018). The target of the World Health Organization was to eliminate Trypanosomiasis as a public health issue by 2020 and to interrupt transmission by 2030 (Franco et al., 2018).

Trypanosomiasis in domestic animals caused by *Trypanosoma brucei*, *T. equiperdum* and *T. evansi* has a huge socio-economic influence and globally decreases reproductive efficiency (Desquesnes et al., 2013). Infection with *T. evansi* has been documented in horses, camels, donkeys and mules in various Asian, African, South American and European countries (Aregawi et al., 2019) and this trypanosome is now considered as an emerging zoonotic parasite (Fong 2017).

To control trypanosomiasis, researchers are seeking to find some alternative medications especially from natural sources where the currently used drugs possess side effects (Kirchhoff 2009). Many herbal extracts like *Azadirachta indica*, *Acacia albica*, *Achyrocline satureioides* and *Indigofera oblongifolia* (Dkhil et al., 2020) are safe effective anti-parasitic agents.

In this study, we used *T. evansi* as a blood parasite model for trypanosomiasis induced in mice. The garlic, *Allium sativum* was used as the potential anti-parasitic agent. *A. sativum* belongs to family Liliaceae and it had been considered as a good agent with antioxidant activity and used for the treatment of heart diseases, hypertension and cancer (Lanzotti, 2006, Suleria et al., 2015, Van Wyk and Wink, 2015). Also, the antimicrobial (Li et al., 2015), the anticoccidial (Dkhil et al., 2011) and the anti-trypanosomal (Krstin et al. 2018) effect of *A. sativum* has been documented. In addition, it has anti-trypanosomal effect (Krstin et al. 2018). The aim of this research was to evaluate the anti-trypanosomal, antioxidant and hepatoprotective role of *Allium sativum* extract (ASE) in mice.

## 2 Materials and methods

### 2.1 Preparation of garlic for treating animals

Fresh *A. sativum* extract (ASE) was prepared by homogenizing 100 g cloves of garlic purchased from the local market, Riyadh, Saudi Arabia. In brief, garlic was cut into small pieces, homogenized in distilled water for 2 min at a final concentration of 20 mg/ml. The debris was removed by centrifugation at 1000 g for 10 min. The supernatant was then rotary-evaporated by Yamato RE300 rotary vacuum evaporator (Tokio, Japan) at 39 °C (Shirzad et al., 2011).

### 2.2 Infrared spectroscopy

Sigma-Aldrich table (www.sigmaaldrich.com/technical-documents/articles/biology/ir-spectrum-table) was used to estimate the expected classes of compounds of *A. sativum*. In brief, ASE was mixed with potassium bromide powder (1: 99 wt%) to obtain a translucent sample disc. The NICOLET 6700 Fourier-transform Infrared Spectroscopy (FT-IR) optical spectrometer from Thermo Scientific (Waltham, MA, USA) was used for the analysis.

### 2.3 Infection and treatment

Male C57BL/6 mice (8–10 weeks old) from the animal facility at Zoology Department were used as experimental animals. Mice were kept in clean cages under standard illumination conditions with a 12-h light–dark cycle and 50% humidity at 25 ± 2 °C. Animals were given a normal diet and water ad libitum. Mice were infected with crypopreserved *T. evansi* and then weekly passaged with infected blood with *T. evansi*. A collected blood drop from the tail vein of mice infected with *T. evansi* was used to determine parasitemia (Herbert and Lumsden 1976). Forty mice, with ten animals per group, were split into 4 groups. The non-infected control group were daily gavaged with distilled water for four days. The second group was treated with 100 µl ASE (20 mg/kg) via oral route (Mikail 2009) while the third and the fourth group were intraperitoneally infected with 1000 *T. evansi*. Mice of ASE-treated group (the fourth group) were orally treated with ASE (20 mg/Kg) 1 h after infection (daily for four days) (Dkhil et al., 2019). Animals were sacrificed by CO_2_ asphyxiation to collect Blood and liver tissue on day 4 postinfection. All experimental animals at Helwan University meet with the National Health Institute Guide for the treatment and use of scientific research.

### 2.4 Hematological study

Blood was gathered into heparinized tubes from the hearts of mice. To measure total leukocytes and erythrocytes and hemoglobin content, an automated counter (VET-530 CA Medonic; Medonic, Stockholm, Sweden) was used.

### 2.5 Liver histology

According to Drury and Wallington (1980), liver pieces were fixed in 10% formalin and then processed to be embedded in paraffin and 5 µm sections were obtained. Finally, sections were stained with hematoxylin and eosin (Drury and Wallington 1980).

### 2.6 Oxidative status

To determine the concentration of the oxidative stress markers, the liver homogenate was prepared (Tsakiris et al., 2004). The concentration of glutathione, malondialdehyde and catalase in the liver were determined according to Ellman, 1959, Ohkawa et al., 1979, Aebi, 1984, respectively.

### 2.7 Statistical evaluation

One-way analysis of variance was used and statistical comparisons were done using Duncan’s test. Data were expressed as mean and standard deviation at p ≤ 0.05 by using SigmaPlot 2011 (Systat Software, Inc., Chicago, IL, USA).

## 3 Results

**Fig. 1:**
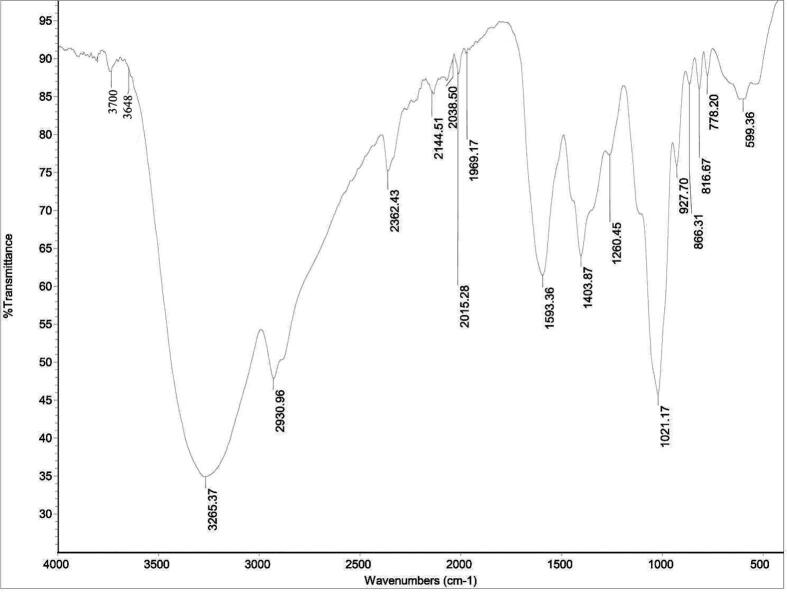
Infrared spectroscopy of *Allium sativum* extract.

ASE exhibited bands between 3700 cm^−1^ and 599 cm^−1^ (Fig. 1, table 1). In the spectrum (Fig. 1) the most relevant bands are those observed at 3265, 1593, 1403 and 1021 cm^−1^, corresponding to the O-H, N-O, S = O and C-N, respectively. Other specific bands ascribed to thiocyanate at 2144 cm-1 and isothiocyanate at 2038 cm^−1^.

**Table 1:** IR spectrum of *Allium sativum* extract by frequency range.

Absorption (cm^−1^)	Appearance	Transmittance (%)	Group	Compound class
3700–3648	Medium	89.6–88.8	O-H stretching	alcohol
3265.73	Strong	34.9	O-H stretching	alcohol
2930.96	Medium	47.8	C-H stretching	alkene
2362.43	Medium	75.1	P-H	phosphine
2144.51	Strong	85.8	S-C = N stretching	thiocyanate
2038.50, 2015.28	Strong	90–88.3	N = C = S stretching	isothiocyanate
1969.17	Medium	90.9	C = C = C stretching	allene
1593.36	Strong	61.4	N-O stretching	nitro compound
1403.87	Strong	64.1	S = O stretching	sulfate
1260.45	Strong	77.3	C-O stretching	alkyl aryl ether
1021.17	Medium	45.8	C-N stretching	amine
927.70	Strong	75.8	=C-H	alkene
866.31; 816.67	Medium	86.6; 86.1	C = C bending	alkene
778.20	Strong	87.8	C-H bending	1,2,3-trisubstituted
599.36	Strong	84.6	C-Br stretching	halo compound

On day 4 post *T. evansi* infection, ASE was able to suppress the parasitemia by 91.5% (Table 2). The number of leucocytes and erythrocytes decreased in the blood of the infected mice compared to the non-infected community. However, after treatment with ASE, the number of leucocytes increased to reach 6.3 ± 0.5 × 10^9^ mm^−3^ and 9.1 ± 1.5 × 10^12^ L^-1^, respectively (Table 2). In addition, ASE significantly increase the hemoglobin content in the infected mice blood (Table 2).

**Table 2:** Effect of *Allium sativum* extract (ASE) on Parasitemia, the count of leucocytes and erythrocytes and the content of hemoglobin of mice infected with *T. evansi*.

Group	Parasitemia suppression (%)	Leucocytes × 10*^9^* mm^−3^	Erythrocytes × 10*^12^* L^-1^	Hemoglobin (g dL^-1^)
Control	–	6.0 ± 1	8.8 ± 1	12.8 ± 2
ASE	–	6.2 ± 0.4	7.7 ± 1.4	11.6 ± 2
Infected	0	4.8 ± 1.0*	6.3 ± 1*	9.4 ± 1*
Infected + ASE	91.5 ± 4^#^	6.3 ± 0.5^#^	9.1 ± 1.5^#^	13.8 ± 2^#^

Values are mean ± SEM, * (significance against control), # (significance against infected) are significance at p ≤ 0.05.

**Fig. 2:**
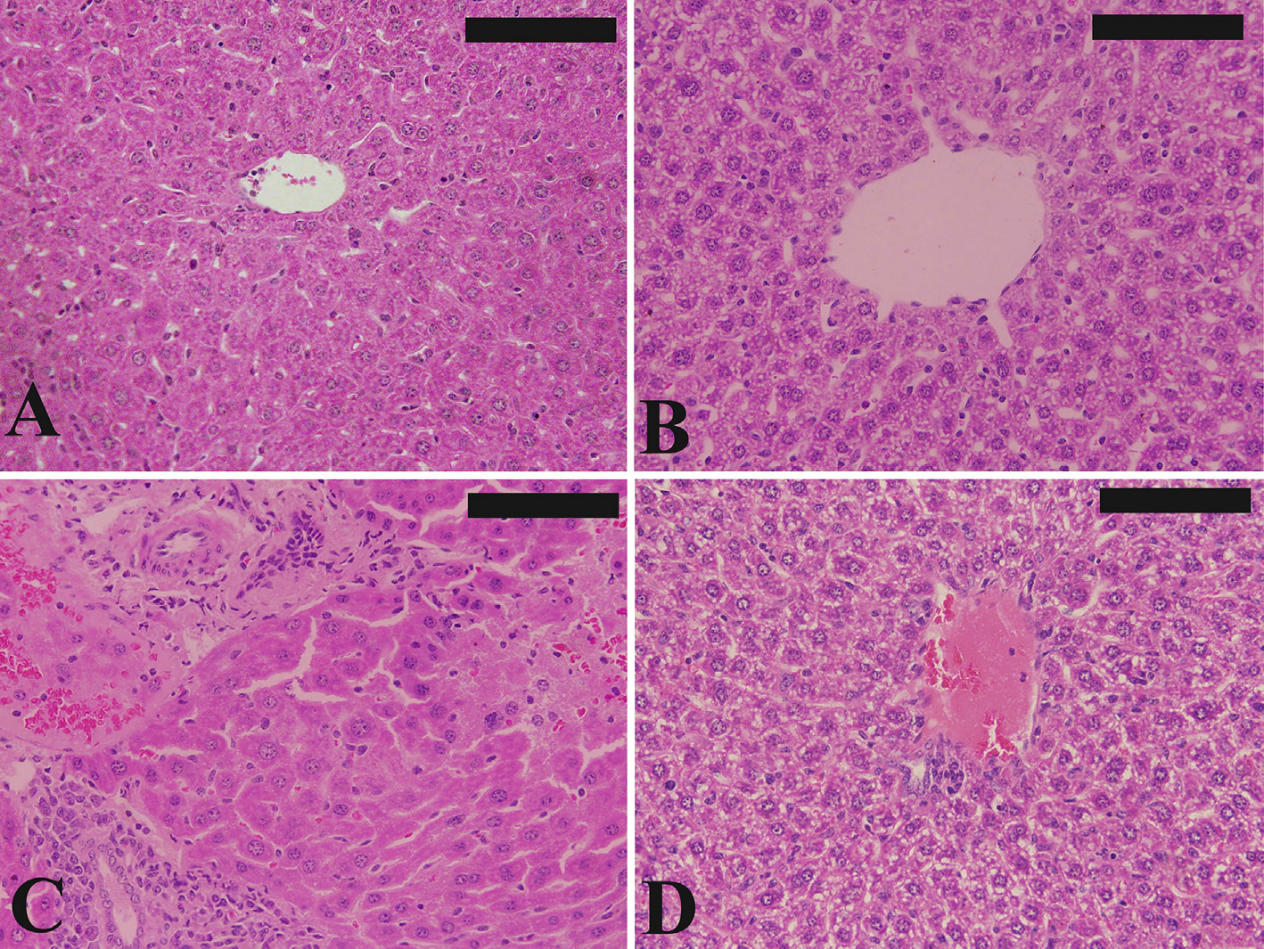
Effect of *Allium sativum* extract (ASE) on liver histology of mice infected with *T. evansi*. Non-infected (A) and infected ASE-treated (B) liver with normal liver structure. The infected mice liver section contained inflammatory cells, sinusoidal dilatation, hemorrhage and increased number of Kupffer cells (C). The infected-treated liver sections were improved (D). Bar = 50 µm.

The infected liver sections appeared with marked changes in the form of inflammation, sinusoidal dilatation, Kupffer cell hyperplasia and the presence of trypanosomes in the central vein. Improvement in the liver structure of the infected treated group has been observed (Fig. 2).

To determine the oxidative status during infection and after treatment of animals with garlic, the level of glutathione was estimated. The *T. evansi* infected liver contained decreased glutathione (3.4 ± 0.31 mg dL^-1^) level compared to the non– infected control liver (5.13 ± 0.51 mg dL^-1^). Also, the catalase activity decreased in the infected animals (3.9 ± 0.4 U g^−1^). ASE could increase the level of glutathione and the activity of catalase in the infected mice (Table 3). However, the level of malondialdehyde was decreased after treatment of animals with ASE to reach 147 ± 1 nmol g^−1^ (Table 3).

**Table 3:** Effect of *Allium sativum* extract (ASE) on hepatic oxidative stress resulted from trypanosomes infection.

Groups	Glutathione (mg dL^-1^)	Malondialdehyde (nmol g^−1^)	Catalase (U g^−1^)
Control	5.13 ± 0.51	165 ± 11	5.5 ± 0.92
ASE	8.4 ± 0.29	153 ± 3	4.7 ± 0.33
Infected	3.4 ± 0.31 *	204 ± 8 *	3.9 ± 0.4 *
Infected + ASE	6.13 ± 0.25 ^*#^	147 ± 1^*#^	5.4 ± 0.7^#^

Values are expressed as means ± SD. *: Significant against control group at p ≤ 0.05, ^#^: Significant against infected group at p ≤ 0.05.

## 4 Discussion

Trypanosomiasis research helped for the reduction of the induced infection where the world health organization documented that between 1999 and 2019, the reported number of new cases of the human African trypanosomiasis, *T. b. gambiense* fell by 97%, and that of *T.b. rhodesiense* fell by 81% (WHO 2019).

Awareness, management steps and studies into enhanced control tools have been seriously neglected in view of the economic and animal health impacts of trypanosomiasis (Birhanu et al., 2016). However, an increasing number of research studies have recently been conducted into the prevalence and control of *T. evansi* infection in animals (Aregawi et al., 2019).

Since anti-trypanosome drugs cause toxicity to the host (Do Carmo et al., 2015), researchers are seeking to find a safe source to control the disease. *Allium sativum* is one of the most effective natural products against parasites (Krstin et al., 2018). The in vitro (Lun et al., 1994) and in vivo (Rossi et al., 2013) antitrypanosomal effect of garlic had been previously reported but still the mechanism of garlic action is unknown. As expected, the IR analysis of the ASE showed the presence of sulfur compounds, that may be as allicin and ajoene (Krstin et al., 2018), to which the biological activity of garlic has been attributed. In our IR results (Table 1), garlic contained sulfate containing group at 1403 cm-1 which may be allicin. Moreover, thiocyanate and isothiocyanate as expected compound class in ASE were reported to functions in host defense against microbes in addition to their antioxidant activity (Chandler and Day, 2012, Mahn and Castillo, 2021).

The infection induced decrease in erythrocytes and hemoglobin content is an indication for anemia. Suliman and Feldman (1989) reported that anemia is a major symptom of the trypanosome infection. Also, Al-Otaibi et al. (2018) reported that the infection with *T. evansi* was associated with lymphocytopenia. Moreover Dkhil et al. (2019) related the decrease in white blood cells during infection to the induced spleen damage.

It is understood that toxins released into the plasma and tissues by the parasite may play a significant role in histopathological liver changes (Ghaffar et al., 2016) that may lead to initiate cellular necrosis (Biswas et al., 2001).

*A. sativum* containing compounds have anti-oxidative and free radical scavenging properties and could modulate oxidative stress (Ademiluyi et al., 2013). Furthermore, garlic attenuated hepatotoxicity effect of nitrate in rats and may decrease lipid peroxidation and improve antioxidant status (El-Kott, 2012). In this research, parasite infection has considerably affected the hepatic oxidative status. Here, the ASE antioxidant effect in the hepatic tissue was demonstrated via regulations of glutathione and catalase. This was due to the change in the oxidative products represented in malondialdehyde (Wolkmer et al., 2009). The findings presented are consistent with those stated by Dkhil et al., (2020), by reporting the antioxidant activities of *Indigofera oblongifolia* extract in the liver against the oxidative damage induced by *T. evansi*.

## 5 Conclusion

Based on our results, *A. sativum* could be used as anti-trypanosomal and antioxidant agent protecting the liver from the infection induced damage but further studies are required to investigate the mechanism of action of the fractionated components of *Allium sativum*.

## Declaration of Competing Interest

The authors declare that they have no known competing financial interests or personal relationships that could have appeared to influence the work reported in this paper.
